# Triage of HPV-positive women in Norway using cytology, HPV16/18 genotyping and HPV persistence

**DOI:** 10.1038/s41416-020-0787-9

**Published:** 2020-04-03

**Authors:** Marc Arbyn, Remila Rezhake, Susan Yuill, Karen Canfell

**Affiliations:** 1 Unit of Cancer Epidemiology, Belgian Cancer Centre, Sciensano, Brussels, Belgium; 2 Department of Cancer Epidemiology, National Cancer Center/National Clinical Research Center for Cancer/Cancer Hospital, Chinese Academy of Medical Sciences and Peking Union Medical College, Beijing, China; 3 Cancer Research Division, Cancer Council NSW, Sydney, Australia; 4 School of Public Health, University of Sydney, Sydney, Australia

**Keywords:** Diagnostic markers, Health policy

## Abstract

In a Norwegian pilot, triage of high-risk human papillomavirus (hrHPV)-positive women with reflex cytology followed by hrHPV testing 12 months later, yielded 82% of women referred to colposcopy and 24% with CIN3+. A policy stratified by the presence of HPV16/18 would be more efficient (66% referred to colposcopy) at the expense of small losses in the detection of precancer.

## Main

Since HPV (human papillomavirus)-based cervical cancer screening is more effective than cytology-based screening,^1,2^ more and more countries are switching to virological testing. As HPV assays have a lower cross-sectional specificity compared with microscopic inspection of Pap smears, appropriate triage of high-risk HPV (hrHPV)-positive women is crucial. Four interacting aspects should be considered when defining a triage policy: (i) the underlying risk of having present or incipient precancer (determined by current screen test results and previous screening history, HPV vaccination status, age and possibly other characteristics), (ii) the triage test or a combination of tests, (iii) choice of thresholds for subsequent management of triage-positive and -negative women, and (iv) adherence to follow-up recommendations. In 2015 European guidelines, two triage procedures are recommended: reflex cytology followed by repeat cytology or by hrHPV retesting if reflex cytology is negative.^3^ Avoiding HPV screening in unvaccinated women younger than 30 years (where HPV infection is common and usually transient), adherence to recommended triage protocols and longer screening intervals (for instance 5 instead of 3 years) can reduce the number of unnecessary referrals and treatments, and the inherent risk of obstetric morbidity.

In the current issue of the *British journal of Cancer*, Hashim et al. described the first triage findings among women included in the HPV screening pilot cohort, implemented in four Norwegian counties.^4^ Women who were hrHPV-positive in the experimental cohort were triaged by reflex cytology, and if the result was cyto-negative, retested for hrHPV 12 months later. Those who were positive at reflex or delayed triage (i.e. at 12-month follow-up) were referred to colposcopy and biopsy, whereas those who were negative at all immediate and delayed triage tests were referred back to routine screening 5 years later. The HPV screening assay used was the cobas 4800 (Roche), which allows identification of HPV16, HPV18 and the bulk of 12 other hrHPV types together. While management was not influenced by HPV genotyping, the performance of an alternative triage based on the combination of genotyping and cytology could be estimated by linking the cobas 4800 data with screening, and pathology registries with comprehensive data capture. A strength of the study is the linkage to a comprehensive registry that allows monitoring of the early effects on the new HPV screening programme, and provides ongoing evidence to inform about possible future adjustment of triage policies for risk-based management.

At reflex triage, 56% of hrHPV+ women with known cytology (1604/2882) showed ASC-US or worse, of whom 612 were found to have cervical precancer (defined as intra-epithelial neoplasia of grade 3 or worse [CIN3+]), corresponding to a positive predictive value (PPV) of 38%. Among 1278 women with normal reflex cytology, 1056 participated at the second triage 12 months later (compliance of 83%), and 626 of them still tested hrHPV+ (persistence of 59%), with a further 88 CIN3+ cases identified. The PPV for CIN3+ of persistent hrHPV among women with normal reflex cytology was 14%. If subjects with unknown or unsatisfactory cytology (for whom no clear follow-up data were presented) are excluded, and adjusting for incomplete delayed triage compliance, it is estimated that ~82% of hrHPV+ patients were referred for colposcopy, resulting in a cumulative detection of CIN3+ of 25%. The estimated number of colposcopies needed to detect one CIN3+ (NNC) case was 3.18. The risk of CIN3+ among women who were twice triage-negative (at baseline and at 12 months) could not be assessed since they were released from follow-up, and no data were available from the next screening-round data yet.

As expected, women with HPV16/18 had higher risks of CIN3+ than women with other hrHPV infections. In the reflex ASC-US+ group, with known typing data, 527 were HPV16/18+ and 1017 were other hrHPV+. However, in the former group, 375 CIN3+ (PPV 71%) were found versus 270 CIN3+ in the latter (PPV 27%). Based on these findings, an alternative algorithm was proposed to triage hrHPV+ women in Norway, with reflex cytology at cut-off ASC-US in HPV16/18+ women versus a cut-off ASCH/AGC+ (atypical squamous cells where high-grade intra-epithelial lesions cannot be excluded/atypical glandular cell, or worse) in other hrHPV+ women. Delayed triage with hrHPV testing at 12 months is then proposed for women with HPV16/18+/NILM and other hrHPV+/ASC-US-LSIL, and at 24 months for women with other hrHPV+/NILM. At the level of reflex triage, this algorithm would result in 796 colposcopies and 532 CIN3+ cases. At delayed triage, assuming complete compliance, we estimate 1110 more colposcopies and 148 CIN3+. Finally, over the two triage rounds, the estimated colposcopy referral rate might be 66% with a CIN3+ detection rate of 24%.

To conclude, the new Norwegian triage algorithm would be slightly more efficient (NNC of 2.81 versus 3.18) at the expense of some cases left undetected (cumulative CIN3+ detection in hrHPV+ women of 24% instead of 25%). A disadvantage of the newer triage strategy might be the higher level of complexity and the longer duration of follow-up for women with other hrHPV/NILM, which might result in a higher probability of dropping out.

**Fig. 1:**
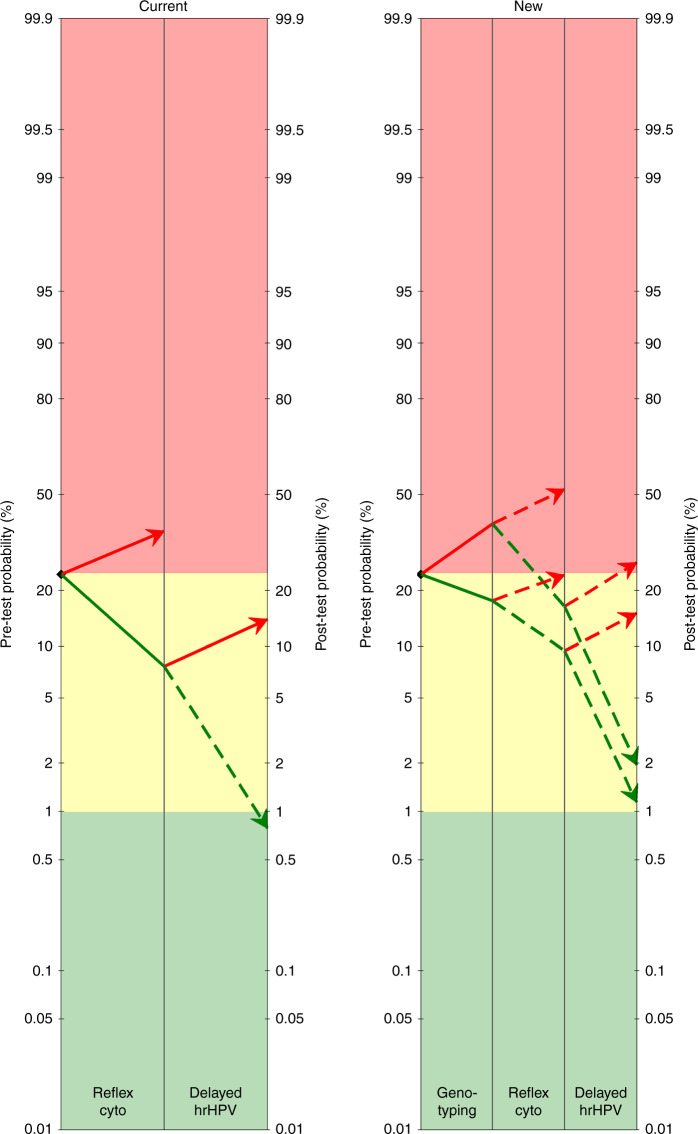
Pre-test and post-test probability (ppp) plots displaying the risk of CIN3+ among hrHPV+ women triaged according to current (left) or new algorithms in Norway. Risks >24% should trigger referral to colposcopy/biopsy (red zone), risks <1% suggest release to routine screening (green zone) and in-between risks suggest further surveillance with repeat testing (yellow zone). Current algorithm: Reflex cytology at cut-off ASC-US and hrHPV testing 12 months later if reflex cytology shows NILM. Women with ASC-US+ at reflex triage or persistent hrHPV+ at delayed triage are referred to colposcopy. Women who cleared hrHPV are released to routine screening. New algorithm: HPV16/18 genotyping followed by cytology. Reference to colposcopy if HPV1618+ & ASC-US+, or other hrHPV+ & ASCH+. Women are referred to delayed triage with hrHPV testing if HPV1618+ & NILM, or other hrHPV+ & ≤LSIL. Those with persistent hrHPV are referred to colposcopy and those who cleared hrHPV are released to routine screening. This figure has been produced using data from refs. ^4^ and ^8^.

The utility of a test or a combination of tests in a given setting can be easily visualised in a pre-test and post-test probability (*ppp*) plot, where the general risk before triage is plotted on the left, and the risk according to the triage results on the right.^5–7^ When the risk of CIN3+ in triage-positive women is higher than an agreed decision threshold (red zone in the *ppp* plot), diagnostic workup with colposcopy and biopsy is indicated. Hashim et al. proposed the decision threshold triggering referral to be at 24% for Norway. The *ppp* plot in Fig. 1 shows the post-test risks expected after application of the current and new Norwegian triage algorithms. The risks were computed by applying the observed cumulative CIN3+ risk (~24%) over a period of 21 months, the observed accuracy for the reflex testing completed with accuracy estimates of delayed triage derived from a recent meta-analysis.^8^ The current algorithm shows that normal cytology and hrHPV clearance reduces the risk sufficiently to allow release to normal screening. However, normal cytology and persistent hrHPV corresponded with a PPV of 14%, which is lower than the Norwegian 24% threshold, but would trigger referral according to most guidelines with lower decision thresholds.^5,6,9^ It must be noted that the overall CIN3+ risk among hrHPV women observed in the Norwegian cohort was among the highest in the triage literature.^8^ Higher rates of CIN3+ are expected in the first screening round after a transition from cytology- to HPV-based screening due to the higher test sensitivity of the latter screening; however, some level of overdiagnosis cannot be excluded.

These findings suggest consideration, at least in some settings, of the possibility of increasing the follow-up time before assessing persistence (e.g. 24 versus 12-month follow-up); although resulting in some detection loss, this will allow a greater proportion of transient infections to clear, and has potential to lead to greater efficiencies in identifying and referring at-risk women with persisting infections. As HPV-based primary screening programmes continue to evolve, a key area where studies such as the present one will contribute to generating real-world evidence regarding the optimal follow-up time for assessing clearance of hrHPV infection and the colposcopy referral criteria at follow-up.

The authors rightly make the point that the use of partial genotyping in primary HPV-based cervical screening programmes enables better balancing of the benefits of screening versus the referral rates to colposcopy, since women with intermediate-risk ‘other hrHPV type’ infections can be managed differently to those with higher-risk HPV16/18 infections. Furthermore, partial genotyping-based colposcopy referral strategies can be explicitly designed to take into account the rapidly dropping prevalence of HPV16/18 in young women due to population-level impact of HPV vaccines. Such an approach is already being used in the National Cervical Screening Program in Australia where due to vaccination, HPV16/18 infection rates are now ~2% or less, even for women in their twenties, which is enabling the practical implementation of HPV screening and immediate colposcopy referral for all HPV16/18-positive women from the age of 25 years,^10,11^ whereas women with other hrHPV infections are triaged with cytology. As HPV-based primary screening programmes around the world evolve and are adapted to the latest evidence, partial genotyping is expected to increasingly find its place as a key strategy for maximising the benefits, and minimising the harms, of cervical screening.

## Data Availability

Not applicable.
